# Critical experimental parameters related to the cytotoxicity of zinc oxide nanoparticles

**DOI:** 10.1007/s11051-014-2440-0

**Published:** 2014-05-20

**Authors:** Yan Zhang, Kathy C. Nguyen, David E. Lefebvre, Phillip S. Shwed, Jennifer Crosthwait, Genevieve S. Bondy, Azam F. Tayabali

**Affiliations:** 1 Mechanistic Studies Division, Environmental Health Science and Research Bureau, Health Canada, 50 Colombine Driveway, Ottawa, ON K1A 0K9 Canada; 2 Regulatory Toxicology Research Division, Bureau of Chemical Safety, Health Canada, 251 Sir Frederick Banting Driveway, Ottawa, ON K1A 0K9 Canada

**Keywords:** Zinc oxide nanoparticles, Characterization, Cytotoxicity, Intracellular reactive oxygen species, Toxicity, Health and environmental effects, OECD program

## Abstract

The increasing use of zinc oxide nanoparticles (ZnO-NPs) has raised concerns about their potential hazards to human and environmental health. In this study, the characterization and cytotoxicity of two ZnO-NPs products (Z-COTE and Z-COTE HP1) were investigated. The zinc content of Z-COTE and Z-COTE HP1 was 82.5 ± 7.3 and 80.1 ± 3.5 %, respectively. Both ZnO–NP samples contained sub-cytotoxic levels of iron and copper, and silicon was detected from the surface coating of Z-COTE HP1. All samples were highly agglomerated, and the primary particles appeared as variable polyhedral structures. There was no significant difference in size distribution or average diameter of Z-COTE (53 ± 23 nm) and Z-COTE HP1 (54 ± 26 nm). A dose-dependent cytotoxicity was observed 24 h after exposure to ZnO-NPs, and monocytes were more sensitive than lung epithelial cells or lymphoblasts in both human and mouse cells. There was a significant difference in cytotoxicity between nano- and fine-forms, but only at the threshold cytotoxic dose with cellular metabolism assays. Compared to uncoated ZnO-NPs, the surface coating with triethoxycaprylylsilane marginally attenuated cellular oxidative stress and protected cellular metabolic activity. These results demonstrate the importance of model cell type, dose selection, and cytotoxicity assessment methodology to accurately evaluate the potential toxicity of various nanoparticles in vitro.

## Introduction

Nanoparticles (NPs) are basically defined as particles ranging from 1 to 100 nm in at least one dimension (Liedl et al. 2010). Rapid advances in nanotechnology have resulted in many types of NPs with diverse chemical and physical properties compared to particles in their bulk form, providing opportunities for the development of new applications. In particular, manufactured zinc oxide nanoparticles (ZnO-NPs) are among the most commonly utilized nanomaterials, with applications in biotechnology and consumer products. Some examples are ceramics manufacture, paint formulation, food packaging, sunscreens, and hair care products (Osmond and McCall 2010). The increased production and use of ZnO-NPs enhances the probability of exposure from occupational and environmental settings, as well as consumer products. This enhanced probability has raised concerns regarding their potential for unintended adverse health effects. The extremely small size of NPs increases the possibility of their uptake by cells and interactions with biological molecules and tissues, thereby not only providing advantageous opportunities but also potential risks for their applications. Hence, it is imperative to rigorously characterize the environmental health and safety aspects of human exposure to ZnO-NPs. However, the toxicity research of ZnO-NPs is far behind nanotechnology advances.

Only a few studies have investigated the toxicity of ZnO-NPs on mammalian cells, and these studies have produced conflicting results with respect to cytotoxicity. Gojova and colleagues investigated the cytotoxicity of ZnO-NPs with lengths of 100–200 nm and diameters of 20–70 nm to show that about 50 % of human aortic endothelial cells died after being incubated with ZnO-NPs at a concentration of 50 ppm for 4 h (Gojova et al. 2007). Similarly, using mouse neuroblastoma cells as the model, Jeng and Swanson found that 50 nm ZnO-NPs at a dose of 100 ppm caused 50 % cell death after an exposure for 24 h (Jeng and Swanson 2006). Brunner and colleagues also found that virtually all human mesothelioma MSTO-211H cells or rodent fibroblast 3T3 cells died after being incubated with 19 nm ZnO-NPs at concentrations above 15 ppm for 72 h (Brunner et al. 2006). In contrast, Reddy and colleagues showed that 13 nm ZnO-NPs were only slightly toxic to human T cells at a concentration of 5 mM (1 mM = 81.39 ppm) after an exposure for 24 h (Reddy et al. 2007). This handful of publications demonstrates that information on toxicity of ZnO-NPs is controversial, and possible mechanisms are not yet identified. Moreover, most studies focused on the effects of particle sizes, and the role of various other properties of particles with respect to observed toxicity remains to be fully understood. In addition, designing suitable assays is hindered by a number of factors, one of which is the huge variation in different manufactured nanomaterials with varying physicochemical properties (composition, size, agglomeration, and others).

Nanotoxicology is an important field of study for the assessment of potential health effects of nanomaterials that are unintentionally generated or are manufactured for specific purposes. It is generally accepted that a comprehensive, accurate characterization of NP size, composition, and surface chemistry is essential for developing our understanding of the parameters that contribute to nanotoxicity. In this article, we have extensively characterized two ZnO–NP manufactured products (uncoated Z-COTE and coated Z-COTE HP1) for insight into the interrelationship between toxicological effects and the particle size, shape, surface coatings, and other physicochemical properties. We assessed the effect of the size and surface coating of ZnO-NPs on cell morphology, cytolysis, and cellular metabolic activity for various cell lines (monocytes, lung epithelial cells and lymphoblasts), which are considered as key cell types for immunotoxicological responses from NPs.

## Materials and methods

### Nanoparticles and their dispersions

The OECD-sponsored ZnO-NPs used in this study, Z-COTE (uncoated) and Z-COTE HP1 (coated with triethoxycaprylylsilane), are commercially available from BASF (Mississauga, Canada). Fine ZnO (bulk control particles, micro-sized) and zinc chloride (ZnCl_2_, a soluble zinc compound) were purchased from Sigma-Aldrich (Oakville, Canada). ZnO suspensions and dispersions were prepared in deionized water of liquid chromatography and mass spectrometry application grade (LC/MS water, Fisher Scientific, Burlington, Canada) for primary particle analysis. Z-COTE, Z-COTE HP1, or fine-ZnO in powder form were transferred into sterile zinc-free glass tube with caps. Conditions for sonication were optimized for various times and power settings. Sonication was routinely performed with an Ultrasonic Liquid Processor (MISONIX, New York, USA) for 15 min at 30 % amplitude in a water bath while continuously cooling the samples with ice during sonication. The final energy was 76,148 J, and the power was 75–85 W. All stock dispersions were adjusted to a final concentration of 100 µg/mL, and then further diluted in either LS/MS water or appropriate medium to the desired concentration. Working dilutions were used freshly.

### Characterization of particles

For inductively coupled plasma optical emission spectrometry (ICP–OES) examination, the samples of Z-COTE, Z-COTE HP1, and fine ZnO were prepared in LC/MS water having a final acid content of 5 % HNO_3_. The metal composition of particles was analyzed by ICP–OES using a Perkin-Elmer Optima 5300DV Emission Spectrometer with axial viewing and modified background correction. Elements of interest were confirmed using a minimum of two analytical wavelengths for each element. Five instrument replicate readings were performed on each analyte for each sample determination. The surface chemistry of particles was assessed by X-ray photoelectron spectroscopy (XPS) with a PHI 5000 VersaProbe spectrometer equipped with multiple channel plates and a focused aluminum monochromatic X-ray source. Data were collected using vendor-supplied software and analyzed using CasaXPS with transmission function corrected area sensitivity factors. Concentrations were calculated by omitting hydrogen and normalized to 100 %.

The particle size distribution (PSD) in LS/MS water dispersions before and after sonication was determined using a Malvern dynamic light scattering (DLS) instrument operating at a wavelength of 633 nm and a scattering angle of 173°. Three replicate measurements were performed for each sample. The PSD of evaporated dispersions was determined by transmission electron microscopy (TEM). The diluted suspensions of particles (1 µg/mL) were deposited onto 300 mesh carbon-coated copper grids. Samples were air-dried in a laboratory biosafety hood at room temperature. TEM grids were examined with a JOEL 1230 TEM operating at an accelerating voltage of 60 kV. Particle sizes were measured from the TEM micrographs using the NIS-Elements BR3.1 software (Nikon, Mississauga, Canada). The particles of evaporated dispersions were also observed on pin stub mounts by LVEM5 scanning electron microscopy (SEM) in back-scattered electrons (BSE) mode at a ~5 kV accelerating voltage. The images were acquired by DI-Microcs software. Atomic force microscopy (AFM) topography examinations were carried out on an Agilent 5500 AFM in non-contact mode with a PPP-NCH probe. The diluted dispersions of particles were deposited onto freshly cleaved mica, and images were acquired and analyzed using Picoview 4.0 software.

### Endotoxin detection

The content of endotoxin within test samples was assessed using the ProGene recombinant factor C (rFC) endotoxin detection assay (Lonza, Mississauga, Canada) according to the manufacturer’s instructions. Briefly, 100 μL of the blank endotoxin standard and samples were added to a 96-well plate. After an incubation of 10 min, 50 μL of rFC working reagent (proenzyme, fluorescent substrate, and buffer) was dispensed to each well, and the fluorescence of each well was read at excitation/emission wavelengths of 380/440 nm after a 1-h incubation. The endotoxin concentration of samples was calculated according to a standard curve generated with *Escherichia coli* endotoxin standard. All samples were assayed in triplicate, and assay range was from 0.01 to 10 EU/mL.

### Cell culture and exposure to ZnO-NPs

In this study, three mouse and three human cell lines were used to test the cytotoxicity of ZnO-NPs. The FE1-Muta™ mouse lung epithelial cell line (FE1-MML) was kindly provided by Dr. Paul White (Health Canada). FE1-MML cells were cultured in a 1:1 mixture of Dulbecco’s Modified Eagle’s Medium (DMEM, 4,500 mg/L d-glucose) and F-12 nutrient mixture supplemented with 2 % fetal bovine serum (FBS), 2 mM l-glutamine, 100 U/mL penicillin G and 100 μg/mL streptomycin sulfate, and 1 ng/mL murine epidermal growth factor (Invitrogen, Burlington, Canada). The other cell lines, murine peritoneal monocytes (RAW 264.7), murine lymphoblasts (LBRM-33), human lung epithelial cells (A549), human monocytic cells (THP-1), and human lymphoblasts (MOLT-4), were obtained from the American Type Culture Collection (ATCC, Manassas, USA). Cells were maintained in the appropriate culture medium based on the ATCC instructions, and exposed at 37 °C, 95 % humidity, and 5 % CO_2_. All culture media, serum, and antibiotics were purchased from ATCC. When the cells had reached 80–90 % confluence, they were either subcultured by serial passage (no more than 8–12 passages), or were used for exposure experiments.

Working solutions of ZnO-NPs were prepared by serially diluting the dispersion of particles in the appropriate culture media. Cells were pre-cultured overnight, and the spent media were gently removed and replaced with fresh media containing ZnO-NPs at a concentration of 3.125, 6.25, 12.5, 25, or 50 µg/mL. Cells were exposed to ZnO-NPs for 24 h. Treatments with either medium only, or same concentration of fine ZnO or ZnCl_2_, were used as the controls.

### Cell morphology examination

Cells were seeded in 12-well plates at a concentration of 1 × 10^5^ cells/mL/well. After a 24 h-exposure, culture media were removed gently and replaced with the Live Cell Imaging Solution (Invitrogen, Burlington, Canada). Treated cells were observed with a Nikon TE300 microscope connected to a Nikon Digital Sight DS-L3 camera.

### Cell viability assessment

Cells were seeded into 96-well plates at a density of 1 × 10^4^ cells per well overnight and were treated with ZnO-NPs in a final volume of 100 µL. Cell cytolysis was measured with a Trypan blue dye exclusion method by using the Countess™ automated cell analyzer (Invitrogen, Burlington, Canada). Cellular metabolic activity was determined by two determinations, the 3-(4,5-dimethylthiazol-2-yl)-2,5-diphenyltetrazolium bromide (MTT) assay and 2-(4-iodophenyl)-3-(4-nitophenyl)-5-(2,4-disulfophenyl)-2*H* tetrazolium salt (WST-1) assay. For MTT assay (Nguyen et al. 2013), culture media were replaced by 90 µL of fresh media with 10 µL of the MTT stock solution (10 mg/mL) in each well, and were discarded after a 2 h-incubation. The cell plates were rinsed with PBS, and cells were lysed with DMSO. Absorbance was measured at 505 nm using a multiwell scanning spectrophotometer (Molecular Devices, Sunnyvale, USA). For WST-1 assay, after cell exposure, 10 µL of the WST-1 solution (Roche, Indianapolis, USA) was added to each well and incubated for 1 h, and then the colored supernatants were measured at 440 nm using the multiwell scanning spectrophotometer. Metabolic activity of the ZnO-treated cells obtained from both assays was expressed as a percentage of media-treated controls. All measurements were done in duplicate in five independent experiments.

### In situ intracellular reactive oxygen species (ROS) detection

The intracellular ROS were assessed by the CellROX oxidative stress reagent (Life Technologies, Carlsbad, USA) according to the manufacturer’s protocol. Briefly, after exposure to ZnO-NPs, the cells in the 12-well plates were washed and then stained with 5 mM of CellROX orange reagent and Hoechst 33342 by adding the probe to the complete medium and incubating the cells for 30 min. The cells were then washed with PBS, and the Live Cell Imaging Solution was added. The stained cells were observed under the Nikon TE 2000 fluorescence microscope and were analyzed by using the NIS-Elements BR3.1 software.

### Statistical analysis

Results were compared by one-way analysis of variance (ANOVA) followed by Dunnett’s multiple comparison test for comparison to the controls. All data were expressed as mean ± standard deviation. A value of *p* < 0.05 was considered statistically significant.

## Results and discussion

### Characterization of ZnO nano- and bulk particles

ICP–OES results provided information on the purity and metal composition of the test particles. The zinc content of Z-COTE, Z-COTE HP1, and fine ZnO was 82.5 ± 7.3, 80.1 ± 3.5, and 80.5 ± 4.8 %, respectively. Most other metals of interest (As, Ca, Cr, Hg, Mg, Mn, Pb) for Z-COTE and Z-COTE HP1 were found to be below the limits of detection, except for Cu (10.2 ± 4.1 ppm) detected in Z-COTE and Cu (6.6 ± 0.6 ppm) and Fe (2.3 ± 0.1 ppm) detected in Z-COTE HP1; no significant amounts of impurities were detected in fine ZnO. The elemental compositions (relative at.%) at the particle surface determined from the XPS data are summarized in Table 1. As expected, silicon was detected in the Z-COTE HP1 due to the triethoxycaprylylsilane surface coating. Both ICP–OES and XPS results indicated no significant differences in the total amount of Zn between Z-COTE, Z-COTE HP1, and fine ZnO.

**Table 1:** Elemental concentration of ZnO–NP surface

	Relative at.%		
	Zn	O	Si
Fine ZnO	25.6	38.6	ND
Z-COTE	21.9	35.3	ND
Z-COTE HP1	27.3	39.7	2.3

Elements not detected above the instrument detection limit were indicated with ND. Concentrations were precise to within ± 10 relative percent

**Fig. 1:**
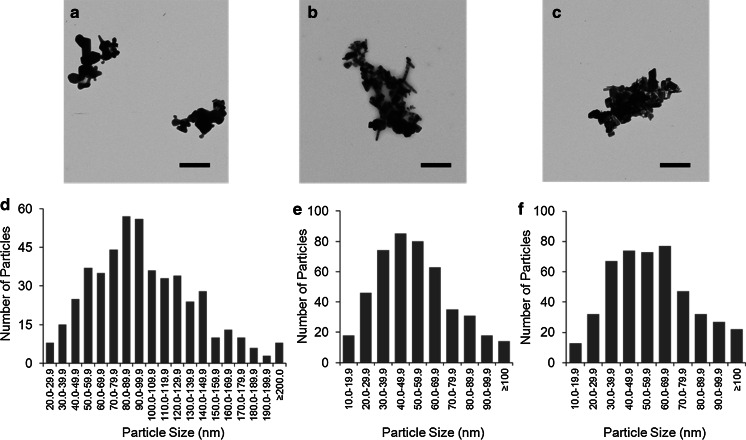
TEM images and size distribution of ZnO-NPs. TEM images: **a** fine-ZnO; **b** Z-COTE; **c** Z-COTE HP1. Each material was suspended in ddH_2_O, sonicated, deposited onto carbon-coated copper grids and allowed to air-dry for 24 h before examination. Z-COTE, Z-COTE HP1, and fine-ZnO appeared as crystalline rods or polygonal structures. Particle agglomeration was observed for all samples. *Scale bar* = 500 nm. Particle sizes were measured from the TEM micrographs of individual particles. Size distribution: **d** Z-COTE (ranging from 12 to 140 nm); **e** Z-COTE HP1 (ranging from 14 to 170 nm); **f** fine-ZnO (ranging from 20 to 300 nm)

TEM images revealed the morphological variation and size distribution of the ZnO crystallites for the evaporated dispersions (Fig. 1). Considerable particle agglomeration was observed in all samples. The primary particles of Z-COTE, Z-COTE HP1, and fine ZnO appeared as hexagonal-based bipyramids, crystalline rods, or elongated polygonal in shape. SEM and AFM images (data not shown) confirmed that evaporated suspensions of Z-COTE, Z-COTE HP1, and fine ZnO were highly agglomerated. Image analysis of the primary particle in the clusters demonstrated that sonication did not significantly alter their size (Table 2). The diameters of primary NPs were measured to be 53 ± 23 nm (ranging from 12 to 140 nm) for Z-COTE and 54 ± 26 nm for Z-COTE HP1 (ranging from 14 to 170 nm). However, the fine ZnO showed a larger average size (106.9 ± 41.1 nm ranging from 20 to 300 nm). In contrast, the DLS measurements for dispersions demonstrated that the particle size of Z-COTE, Z-COTE HP1, and fine ZnO was 336 ± 3, 209 ± 2, and 512 ± 4 nm, respectively. Both DLS and TEM results indicated particle agglomeration for each preparation for all samples, resulting in the formation of flocs of variable sizes from a few hundred nanometers to several microns in diameter (Table 2). Compared to the unsonicated samples, the sonicated samples demonstrated overall smaller particles, which implies that sonication was successful in dissociating the larger ZnO flocs into smaller discrete agglomerates. Preliminary testing with optimized sonication conditions showed that few primary particles were observed under the experimental conditions used.

**Table 2:** Particle size distribution of ZnO-NPs

Sample	Sonication	DLS	TEM			
		Z-average diameter (nm)	Average primary particle size (nm)	Average aggregate size (nm)	Min. aggregate size (nm)	Max. aggregate size (nm)
Fine ZnO	−	633 ± 6	110 ± 44	4,252 ± 3,176	710	8,650
	+	512 ± 4	107 ± 41	1,178 ± 951	100	3,650
Z-COTE	−	945 ± 16	54 ± 25	4,782 ± 3,189	1,210	9,030
	+	336 ± 3	53 ± 23	1,319 ± 1,129	210	4,260
Z-COTE HP1	−	293 ± 1	53 ± 21	5,652 ± 3,067	1,850	10,230
	+	209 ± 1	54 ± 26	1,616 ± 1,139	330	4,850

The chemical and physical state of a nanomaterial are of obvious importance when determining how that material might interact with a biological system. Our study demonstrated that XPS, ICP–OES, DLS, TEM, SEM, and AFM can be used to assess the composition, size distribution, structure, and surface information of ZnO-NPs. We combined these techniques to get more details on characteristics than that provided by the manufacturer. Using ICP–OES, we found copper and/or iron impurities in two commercial ZnO–NP products. It was reported that there was no significant difference in the level of intracellular ROS between control A549 cells and those exposed to 80 µg/mL of Fe_3_O_4_ or 40 µg/mL of CuO, which was 4- to 20-fold greater than what we detected (Karlsson et al. 2008; Mahmoudi et al. 2009). Although these two trace metals were present below levels that would induce any appreciable cytotoxicity, further study might be needed to test the potential synergistic effects with ZnO–NP in toxicity assessment.

DLS was useful for observing trends and comparisons, such as the effectiveness of sonication. Compared to TEM, however, DLS measurements gave a broad range of results and generally lacked reproducibility (Pfaller et al. 2010), especially in the case of a low refractive index material such as ZnO-NPs. Furthermore, DLS has been discouraged in the analysis of highly polydispersed NPs such as what we have observed in this study. The characterization information provided by manufacturer showed that Brunauer–Emmett–Teller (BET) surface of Z-COTE and Z-COTE HP particles was 12–24 m^2^/g and all particle sizes were described as being less than 2 µm. The particle sizes reported by the manufacturer were different from those actually measured in this study. The main reason might be variations in the approaches used for dispersing the preparations, and/or different techniques for measurement and analysis. For example, we used ultra-pure water as a solvent for all characterization work, and the manufacturer employed isopropanol. Moreover, we measured the diameter of the primary particles rather than the length. Even though ZnO-NPs can be easily synthesized, it is difficult to maintain their nano-size in practice. As such, due to their high specific surface area and high surface energy level, NPs have the propensity to agglomerate to form micro-size particles that are more stable in the environment (Heng et al. 2010). This effect was also observed in the present study when ZnO-NPs were suspended in LS/MS water. Both uncoated and coated ZnO-NPs were significantly agglomerated even after sonication, and no nanoscale monomers were present in the TEM or AFM images. Our data using several different physical metrological methods suggest that a multi-method approach is preferable, but the analysis of size distribution by TEM with image analysis software is the most accurate, reproducible, and easiest to interpret.

### Endotoxin contamination of testing samples

An important initial step when characterizing the biological effects of NPs in cell culture systems is the assessment for the presence of endotoxin contamination, which can influence cellular responses. The endotoxin content in ZnO–NP samples was measured using Limulus Amebocyte Lysate (LAL)-based assay. The highest level of endotoxin (0.520 ± 0.130 EU/mL) was detected in the Z-COTE HP1 group at a NP concentration of 100 µg/mL. At our highest exposure concentration (50 µg/mL), the average level of endotoxin was only 0.072 EU/mL (Z-COTE), 0.139 EU/mL (Z-COTE HP1), 0.178 EU/mL (fine ZnO), and 0.102 EU/mL (ZnCl_2_). These levels of endotoxin were relatively low (Vallhov et al. 2006) and when 0.5 EU/mL of lipopolysaccharide (LPS) was added to the cells, no biological parameters tested were affected (data not shown). Morris and colleagues reported that more than approximately 3 EU/mL endotoxin would be necessary to induce cellular immune responses (Morris et al. 1992). However, the values we observed in the administered compounds were far below this level and so we conclude that endotoxin is not a contributing factor in the cytotoxicity observed in the present study, and the cellular responses we detected are caused by the particles.

### Cytotoxicity induced by ZnO-NPs

**Fig. 2:**
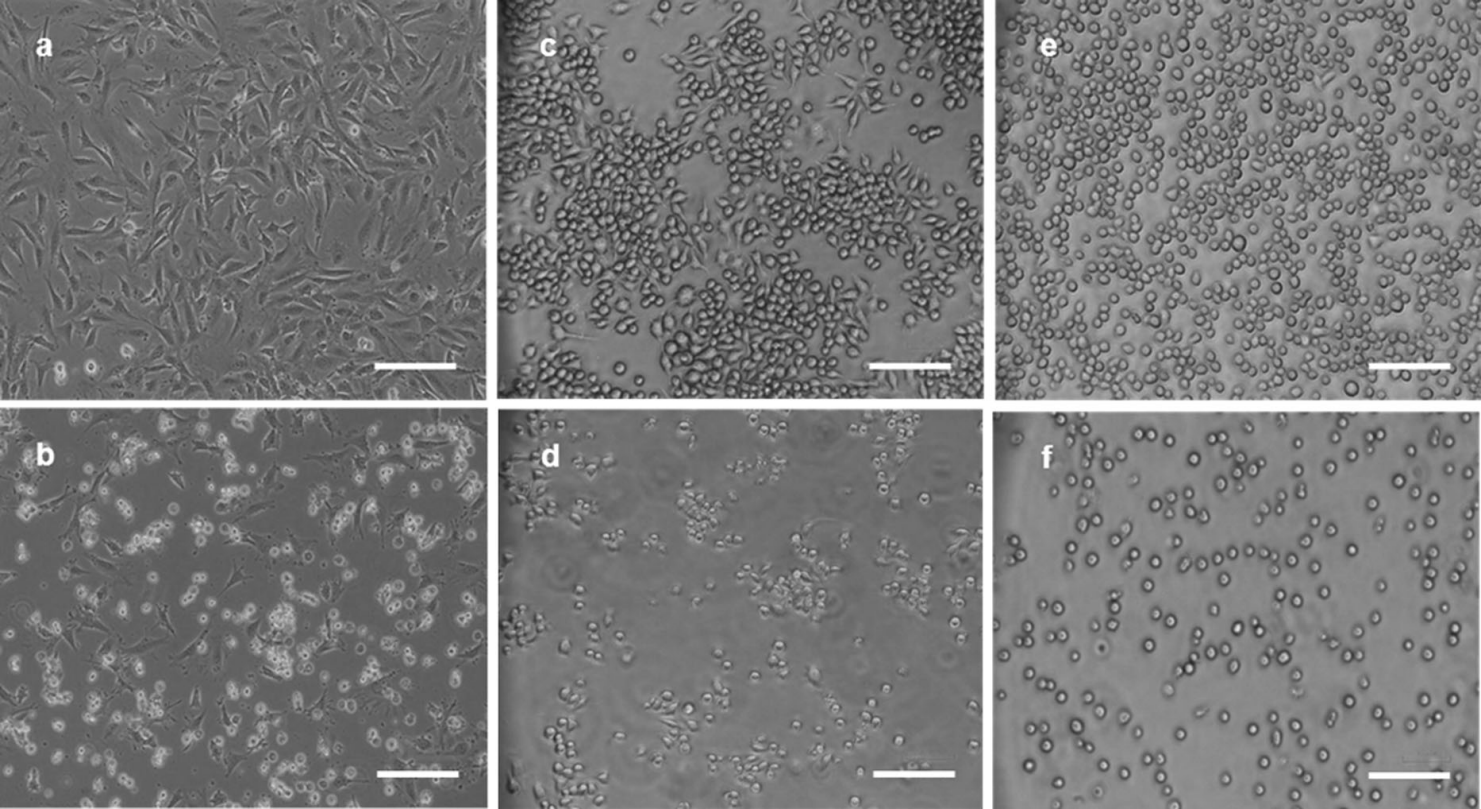
The effect of ZnO-NPs on cell morphology. After ZnO–NP exposure (25 µg/mL) for 24 h, treated cells were observed with a Nikon TE300 microscope. **a**, **b** FE1-MML cells. **a** non-treated cells; **b** Z-COTE-treated cells. **c**, **d** RAW 264.7 cells. **c** non-treated cells; **d** Z-COTE-treated cells. **e**–**f** LBRM-33 cells. **e** non-treated cells; **f** Z-COTE-treated cells. Exposure to a high concentration of Z-COTE altered the gross cell morphology (cell shape and detachment) of all mouse cell lines. *Scale bar* = 100 µm

Our cell culture exposure system included six types of cell lines—mouse and human lung epithelial cells, monocytes, and lymphoblasts. All cell populations were individually exposed to a wide concentration range (0–50 µg/mL) of each particle type. In order to investigate gross cell damage following treatment with ZnO-NPs, bright field microscopy was used to directly view cell morphological changes. No significant adverse effects were observed in cell morphology for any of the cell lines exposed to levels below 6.25 µg/mL of particles at 24 h of exposure. However, dramatic changes in cell morphology (cell shape and detachment) were observed after exposure to ZnO-NPs at the high concentration (above 12.5 µg/mL). Figure 2 shows these changes in three mouse cell lines after exposure to Z-COTE (25 μg/mL).

**Fig. 3:**
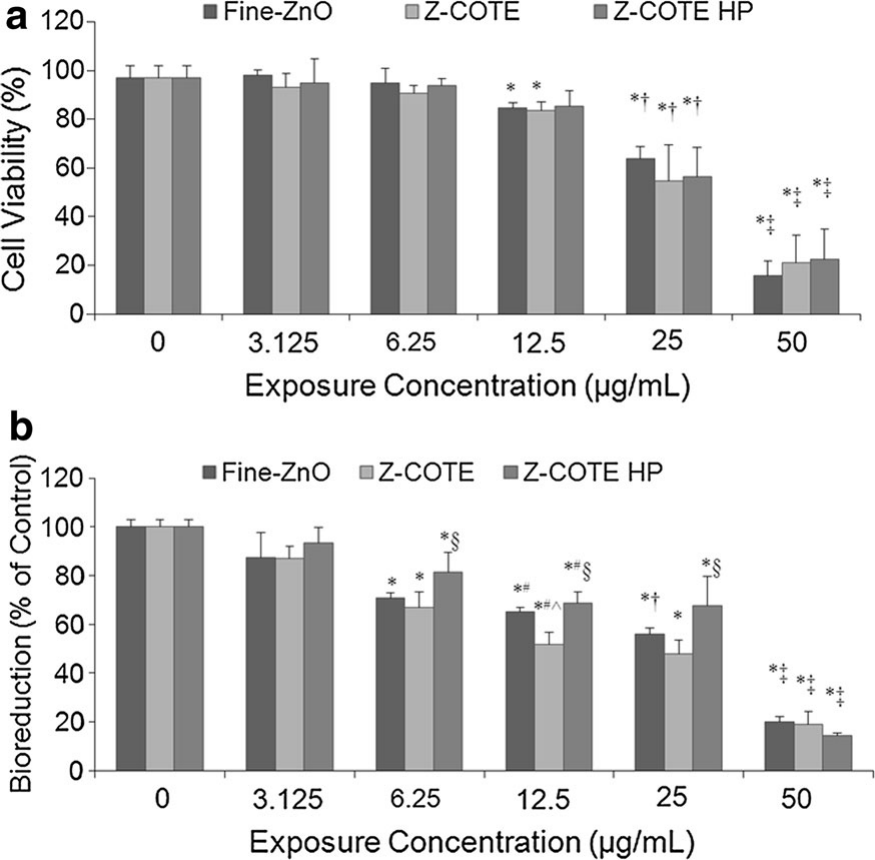
The effect of ZnO-NPs on cell viability. **a** Cytolysis of FE1-MML cells. Cell viability was measured using the Trypan blue dye exclusion method. A Concentration–dependent toxicity was observed after exposure for 24 h. However, there was no significant difference between Z-COTE, Z-COTE HP1 and fine ZnO in cell viability after exposure. **b** Metabolic activity of FE1-MML cells. The MTT assay results confirmed a concentration dependent cytotoxicity (*p < 0.05 vs. control cells; ^#^ *p* < 0.05 vs. 6.25 µg/mL; ^†^ *p* < 0.05 vs. 12.5 µg/mL; ^‡^p < 0.01 vs. 25 µg/mL, *n* = 6), also indicated a slight difference in cellular metabolic activity between the Z-COTE and fine-ZnO and a significant difference in cellular metabolic activity between the Z-COTE and Z-COTE HP1. At a concentration of 12.5 µg/mL, the bioreduction from Z-COTE treatment was higher than that from fine ZnO (^^^ *p* < 0.05 vs. fine ZnO). At a concentration of 6.25, 12.5 or 25 µg/mL, the bioreduction from Z-COTE HP1 treatment was higher than that from Z-COTE (^§^ *p* < 0.05 vs. Z-COTE)

We examined cell cytolysis using the Trypan blue exclusion method, which is able to detect dead cells by way of loss of cell membrane integrity. A dose-dependence of cell cytolysis was observed in FE1-MML cells at 24 h after particle exposures. However, no differences were correlated with particle size or coating at any concentration (Fig. 3a). At the highest concentration (50 µg/mL), ZnO-NPs caused almost all cells to die at the 24 h exposure time. This dose-dependent effect was also observed in other mouse and human cell lines (data not shown).

Some types of NPs can pass through cell membranes and may show unpredictable toxic effects. A toxicological concern arising from this passage is that the NPs could accumulate in mitochondria and affect the mitochondria function (Colvin 2003). Therefore, in this study, mitochondrial function (cellular metabolic activity) was evaluated by MTT and WST-1 assays. The results confirmed the dose-dependent cytotoxicity in all six cell lines. Data shown are for FE1-MML cells (Fig. 3b), but these results are similar to those of all other cell lines. However, unlike the Trypan blue assay, the MTT and WST-1 results indicated a slight but significant difference in cellular metabolic activity between the Z-COTE and fine-ZnO. After exposure with FE1-MML cells for 24 h at a concentration of 12.5 µg/mL, the bioreduction in metabolic activity following Z-COTE treatment (51.8 ± 4.9 %) was significantly lower than that of fine ZnO (65.1 ± 1.9 %, *p* < 0.05).

The parameter of particle size is usually considered when investigating the toxicity of NPs. The biological effects of NP exposure, including toxicity, are often greater than that of micro-scale particles (Limbach et al. 2005). Smaller particles occupy less volume, resulting in a larger number of particles with a greater surface area per unit mass and increased potential for biological interaction (Duffin et al. 2007). Many industrial metal oxide NPs have higher cytotoxic activity than micro-scale particles that have a similar composition formula. However, in this study, the expected overall size-dependent toxicity did not appear; only cellular metabolism assays showed a slight difference between nano- and bulk ZnO treatments at the threshold exposure dose (6.25 µg/mL). One explanation for this observation may be that ZnO particles, whether in the nano- or micro-size range, quickly agglomerated to form larger particles following dispersion in pure water or cell culture medium. Following agglomeration, the net size of bulk and nano--material was essentially the same as shown in Fig. 1. Although our preliminary studies optimized the preparation procedure of ZnO–NP dispersions, all ZnO particles formed aggregates, resulting in larger particle in the micro--range. Therefore, agglomeration and polydispersity should be taken into consideration, as they will influence NP stability and in turn impact the toxicity of particles.

**Fig. 4:**
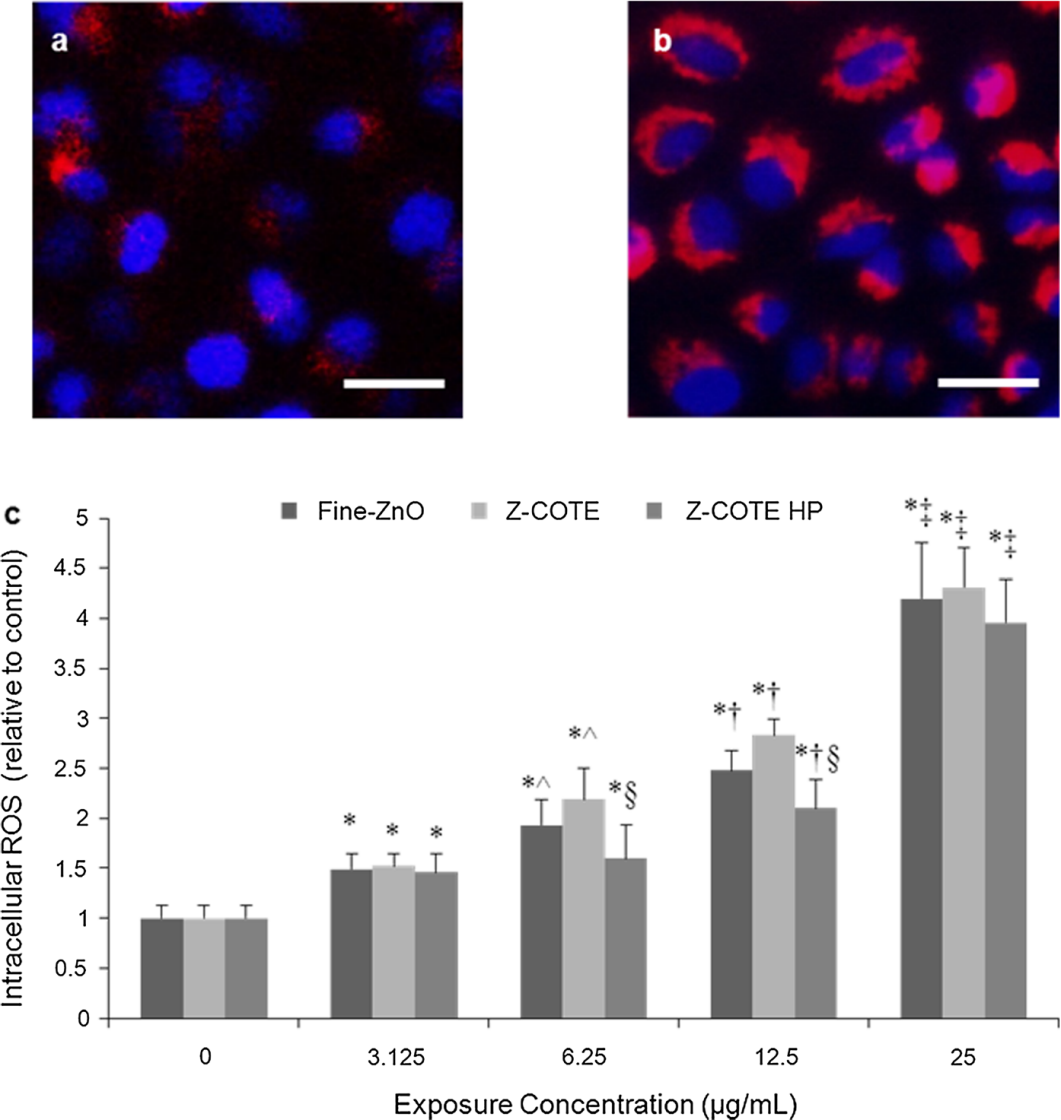
The effect of ZnO-NPs on cellular oxidative stress. **a**, **b** The expression of reactive oxygen species (ROS) in living FE1-MML cells after exposure to even a low concentration of Z-COTE (6.25 µg/mL). **a** non-treated cells; **b** Z-COTE-treated cells. Intracellular ROS was identified by a fluorogenic probe (*red*, cytoplasm) and DAPI (*blue*, nucleus). *Scale bar* = 25 µm. **c** ROS data demonstrated that ZnO-NPs elevated intracellular ROS in a dose-dependent manner (*p < 0.05 vs. control cells; ^^^ *p* < 0.05 vs. 3.125 µg/mL; ^†^ *p* < 0.05 vs. 6.25 µg/mL; ^‡^ *p* < 0.01 vs. 12.5 µg/mL; *n* = 6), and Z-COTE HP1 (surface coating with triethoxycaprylysilane) showed a lower level (^§^ *p* < 0.05, *n* = 6) of ROS compared to Z-COTE (uncoated ZnO-NPs). (Color figure online)

In addition, MTT results indicated a better cellular metabolic activity of FE1-MML cells after exposure with 6.25, 12.5, and 25 µg/mL of Z-COTE HP1 (81.4 ± 7.9, 68.7 ± 4.5, and 67.7 ± 11.9 %, respectively) compared to that of Z-COTE treatment (67.0 ± 6.3, 51.8 ± 4.9, and 48.0 ± 5.5 %, respectively; Fig. 3b). To further investigate whether surface coating with triethoxycaprylylsilane attenuated the cytotoxicity of ZnO-NPs, we measured the intracellular ROS. ROS generation has been considered as one of the common properties of many types of metal-based nanoparticles (NPs) and a major contributor to NP-induced toxicity (Nguyen et al. 2013). At a concentration of 6.25, 12.5, and 25 µg/mL, Z-COTE induced elevated intracellular ROS generation (9–31 %) compared to Z-COTE HP1 (Fig. 4). Previous work has reported that QDs coated with carboxyl groups were less toxic than QDs with an amine surface coating (Slaveykova et al. 2009), indicating that the toxicity of QDs may be controlled by changing their surface chemistry. However, available data on coated ZnO-NPs are often inconsistent (Feltis et al. 2012, Kermanizadeh et al. 2013). Some experimental data reported that there were differences in the cellular association of ZnO-NPs depending on the specific coating used (Heng et al. 2011). Similar results were observed in our study that the surface coating with triethoxycaprylylsilane, marginally, but significantly attenuates the cytotoxic effects of ZnO-NPs. However, this significant difference was not observed in cell morphology and Trypan blue viability between treatments with the two types of ZnO-NPs at any given exposure dose. These results suggested that intracellular ROS examination, and MTT and WST assays might be more sensitive than Trypan blue staining for cytotoxicity assessment of ZnO-NPs. Similar results have also been found in carbon nanotube studies (Monteiro-Riviere et al. 2009). However, it was reported that the use of the MTT or WST-1 assay data may be flawed due to the effects of NPs on the analyte of the detection system (Ng et al. 2005; Ghosh et al. 2010). To address this, we examined the cytotoxicity of ZnO-NPs by using multiple assays, including MTT, WST-1 and Trypan blue assays. The different reporters gave us similar results at our testing dose range (3.125–50 µg/mL). However, above this range, it is possible there may be interaction between dyes and testing platforms related to ZnO particles. Currently, there is no single standardized cytotoxicity method that is ideal for nanotoxicology studies. Use of a combination of assays monitor different cellular structures and functions using varied reagents and measurement systems, limits the probability that an inaccurate measure of cellular health.

**Fig. 5:**
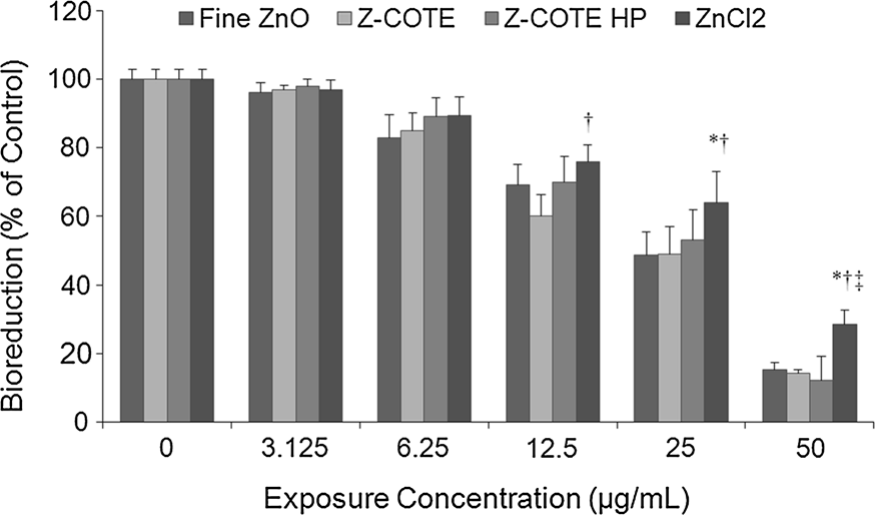
The effect of Zn^2+^ on cellular metabolic activity of FE1-MML cells. ZnCl_2_ had toxic effects on cells. However, this effect was less than that of ZnO-NPs and fine ZnO. (*p < 0.05 vs. fine ZnO; ^†^ *p* < 0.05 vs. Z-COTE; ^‡^ *p* < 0.05 vs. Z-COTE HP1; *n* = 6)

An increase in solubility is another one of the important features of metal oxide NPs, and metal ion release is most important in the cytotoxicity of NPs. The toxicity of ZnO-NPs may be ascribed partly to the dissolved zinc ions in the medium and the released ions might be the primary cause of cytotoxicity. To determine whether the ZnO–NP toxicity differed from that of ionized Zn^2+^, we studied the cytoxicity of ZnO-NPs compared with that of ZnCl_2_ at the same concentration. The MTT results showed that ZnCl_2_ had toxic effects on both human and mouse cells. Figure 5 showed the cellular metabolic activity of FE1-MML cells after exposure to ZnCl_2_. However, this effect was less than (10–50 %) that of ZnO-NPs and fine ZnO, suggesting that free dispersed Zn^2+^ ions that originate from ZnO-NPs may only partly account for ZnO–NP induced cytotoxicity.

Generally, ZnO particles are classified as poorly water soluble. However, ZnO-NPs can release some Zn^2+^ into a culture medium (Hsiao and Huang 2013). Previous investigations suggested that ZnO-NPs showed severe cytotoxicity via Zn^2+^ release (Song et al. 2010; Xia et al. 2008; Yu et al. 2011). It was also reported ZnO-NPs and ZnCl_2_ were comparably toxic to the freshwater microalga and attributed the ZnO–NP toxicity solely to dissolved zinc (Franklin et al. 2007). Another study showed that ZnO dissolution is probable in culture medium, and thus expected that the cultured cells were mainly exposed to aqueous Zn ions when treated with medium to low concentrations of ZnO-NPs (Xia et al. 2008). Our results showed that ZnCl_2_ had an adverse effect on cells. However, the same mass concentration of ZnO-NPs showed a greater effect. Thus, the release of Zn^2+^ from ZnO-NPs might partly induce the cytotoxicity but not be able to fully account for the toxicity mechanism of the NPs. Similar results were also reported in another nanoparticle study (Karlsson et al. 2008). Besides the effect of dissolved ions, it remains unclear what other cellular effect mechanisms may contribute to NP toxicity. One speculation is that cell membranes might be a barrier for Zn ions but the uptake of ZnO-NPs might facilitate the intracellular release Zn ions. This possibility has been described by Limbach and colleagues as a “Trojan-horse” transport and release mechanism (Limbach et al. 2005).

**Fig. 6:**
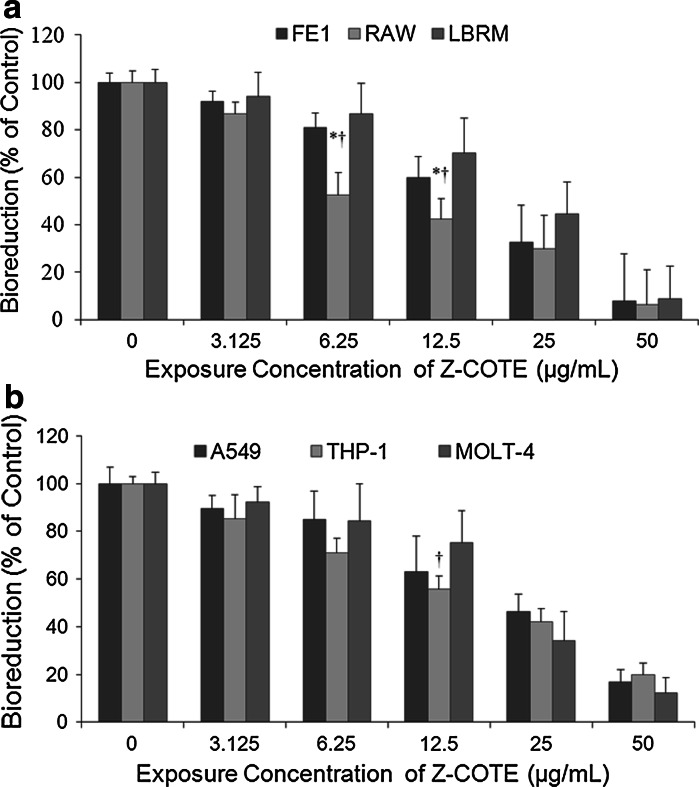
The effect of Z-COTE on different cell types. **a** Cell metabolic activity of mouse cell lines. **b** Cell metabolic activity of human cell lines. The WST-1 assay results confirmed a concentration dependent cytotoxicity in all six cell lines. At the same concentration (12.5 μg/mL), Z-COTE induced a significant variation in cellular viability on monocytes, while no significant toxicity was detected in the epithelial cells and lymphoblasts. In mouse cell lines, the RAW cells were more sensitive than FE1 cells and LBRM-33 cells (*p < 0.05 vs. FE1 cells; ^†^ *p* < 0.05 vs. LBRM cells, *n* = 6). Similar effects were also observed with human cells (^†^ *p* < 0.05 vs. MOLT-4, *n* = 6)

Our cell exposure panel has been designed as a systematic platform for predicting in vivo NP exposure, within a highly controlled and reproducible system, including monocytes, epithelial cells, and lymphoblasts. Monocytes are an important component of immune response, and represent a small but significant proportion of the cells involved in inflammation in the response to a NP insult (Feltis et al. 2012). We expected that this involvement is part of a concerted response involving epithelial cells and those of the lymphocyte lineage. Our WST-1 results indicated that the lowest concentration (3.125 μg/mL) of ZnO-NPs induced a significant variation in cell viability on mouse monocytes, while no significant toxicity was detected in the epithelial cells and lymphoblasts (Fig. 6). Significant differences were observed between cell types at the same exposure conditions (6.25 and 12.5 μg/mL of Z-COTE) in both mouse (Fig. 6a, *p* < 0.05) and human cell lines (Fig. 6b). More specifically, the concentration resulting in the death of 50 % of the mouse monocytic cells (LC was between 6.25 and 12.5 µg/mL), whereas lung epithelial cells and lymphoblast cells were less sensitive, with LC50 values 12.5–25 and 12.5–25 µg/mL, respectively.

There are contradictory reports on the toxicity of particles when various cell lines were exposed to NPs. For instance, toxicity of ZnO-NPs on the human lung epithelial cell line, A549, found that there was no or limited toxicity at the concentrations used (20–80 μg/mL) (Hsiao and Huang 2011). In another study, ZnO-NPs showed severe toxicity at the same concentration with human fibroblasts. However, there was no trace of ZnO–NP toxicity on mouse fibroblast cells even at higher concentrations (500 μg/mL) (Zhang et al. 2011). Our experiments showed that NP cytotoxicity was greater in macrophages than lung epithelial cells and lymphoblasts both in human and mouse cell lines. Uptake by immune cells is likely one of the mechanisms of entry of NPs into cells, but in itself this aspect would not have the capacity to distinguish bulk material from nano-sized particles. As macrophages are actively phagocytic compared to the other two cell types, we speculate that this increased cytotoxicity may have been due to increased uptake of NPs, resulting in greater intracellular exposure to the NPs.

The potential for increased human exposure to ZnO-NPs underlines the need for methodologies to detect adverse effects on human health. However, currently, there are no ideal standardized methods for nanotoxicology studies. By using a combination of the cytotoxicity assays, the various aspects of cell status can be determined with various reagents and measurement systems, essentially minimizing the probability that the NP might interfere with any one system. The cytotoxicity profiles in this study have provided some information on how ZnO-NPs might affect the viability and metabolism of various cellular systems. However, there are more subtle and potentially longer-term consequences of immune cell activation and suppression that must be considered. We are examining these effects in more detail, which will be reported separately.

Selection of concentrations for in vitro treatment as they compare to real-world exposure scenarios is most desirable. Our results indicated the importance of a dose selection for accurate evaluation the potential toxicity of NPs. We noticed that some groups have worked on the dose correlation between the in vitro testing and in vivo exposure (Gangwal et al. 2011). We suggest that the in vivo exposure dose should be selected based on the in vitro results, the commercial product analysis, and some human exposure data.

## Conclusions

We demonstrated that agglomerated ZnO-NPs had toxic effects on mammalian cells, and this effect was dependent on the ZnO concentration and the cell line used. The ZnO–NP toxicity was greater than pure-dissolved Zn^2+^. A slight difference in cytotoxicity between nano- and fine-forms was observed, but only at the threshold dose (12.5 µg/mL) with cellular metabolism assays. Compared to uncoated ZnO-NPs, the surface coating with triethoxycaprylylsilane was observed to marginally attenuate the cellular oxidative stress. These results demonstrate the importance of a model cell type, a dose selection, and cytotoxicity assessment methodology to accurately evaluate the potential toxicity of various NPs. This study has provided insight into some critical parameters for the toxicity of ZnO-NPs and direction for further in vitro and in vivo studies that will be necessary to assess the potential toxicological effect of ZnO-NPs.
